# Race Differences in COVID-19 Stress and Social Isolation: Implications for Depressive Symptoms

**DOI:** 10.1093/geroni/igab046.1978

**Published:** 2021-12-17

**Authors:** Kira Birditt, Akari Oya, Angela Turkelson, Courtney Polenick, Karen Fingerman

**Affiliations:** 1 University of Michigan, Ann Arbor, Michigan, United States; 2 The University of Texas at Austin, Austin, Texas, United States

## Abstract

The experience of the COVID-19 pandemic may vary widely by race. This study examined race differences in pandemic-related stress, social isolation and the implications for well-being. Participants included 1260 adults (45% women) ages 18 to 97 from the May and June 2020 nationally representative Survey of Consumers and 562 who completed a 6 month follow up in November/December. A total of 76% were White, 10% were Black, 3% were Asian, and 11% were Hispanic. Participants reported experiences of pandemic-related stress, social isolation and depressive symptoms in the last month. Analyses showed that minority groups reported greater pandemic related stress that had negative implications for depressive symptoms over time. The implication of social isolation for the stress-depressive symptoms link also varied by race. Overall this study showed racial inequities in the implications of COVID-19 pandemic and that reducing social isolation may only be beneficial for certain racial/ethnic groups.

